# Arg-Leu-Tyr-Glu tetrapeptide inhibits tumor progression by suppressing angiogenesis and vascular permeability via VEGF receptor-2 antagonism

**DOI:** 10.18632/oncotarget.14343

**Published:** 2016-12-28

**Authors:** Yi-Yong Baek, Dong-Keon Lee, Joohwan Kim, Ji-Hee Kim, Wonjin Park, Taesam Kim, Sanghwa Han, Dooil Jeoung, Ji Chang You, Hansoo Lee, Moo-Ho Won, Kwon-Soo Ha, Young-Guen Kwon, Young-Myeong Kim

**Affiliations:** ^1^ Department of Molecular and Cellular Biochemistry, School of Medicine, Kangwon National University, Chuncheon, Gangwon-do, 200-702, South Korea; ^2^ Department of Biochemistry, College of Natural Sciences, Kangwon National University, Chuncheon, Gangwon-do, 200-702, South Korea; ^3^ Department of Pathology, School of Medicine, The Catholic University of Korea, Seoul 137-701, Korea; ^4^ Department of and Life Sciences, College of Natural Sciences, Kangwon National University, Chuncheon, Gangwon-do, 200-702, South Korea; ^5^ Department of Neurobiology, School of Medicine, Kangwon National University, Chuncheon, Gangwon-do, 200-702, South Korea; ^6^ Department of Biochemistry, College of Life Science and Biotechnology, Yonsei University, Seoul, 120-752, South Korea

**Keywords:** antiangiogenic peptide, tumor growth and metastasis, VEGFR-2, VEGF-A

## Abstract

The tetrapeptide Arg-Leu-Tyr-Glu (RLYE) is known to inhibit vascular endothelial growth factor-A (VEGF-A)-induced angiogenesis *in vitro*. Herein, we examined its underlying mechanism and antitumor activity associated with vascular remodeling. RLYE inhibited VEGF-A-induced angiogenesis in a mouse model and suppressed VEGF-A-induced angiogenic signal cascades in human endothelial cells. However, RLYE showed no inhibitory effect on VEGF-A-induced proliferation and migration of multiple myeloma cells expressing VEGF receptor (VEGFR)-1, but not VEGFR-2. In addition, RLYE showed no inhibitory effect on angiogenic activities induced by VEGF-B, basic fibroblast growth factor, epithermal growth factor, sphingosine-1-phosphate, and placental growth factor. RLYE bound specifically to VEGFR-2 at the VEGF-A binding site, thereby blocking VEGF-A-VEGFR-2 binding and VEGF-A-induced VEGFR-2 internalization. The RLYE peptide inhibited tumor growth and metastasis via suppression of tumor angiogenesis in tumor-bearing mice. Moreover, RLYE showed a synergistic effect of the cytotoxic agent irinotecan on tumor cell apoptosis and tumor progression via tumor vessel normalization due to stabilization of VE-cadherin-mediated adherens junction, improvement of pericyte coverage, and inhibition of vascular leakage in tumors. Our results suggest that RLYE can be used as an antiangiogenic and tumor blood vessel remodeling agent for inhibition of tumor growth and metastasis by antagonizing VEGFR-2, with the synergistic anti-cancer effect via enhancement of drug delivery and therapeutic efficacy.

## INTRODUCTION

Robust tumor growth results in hypoxia, which promotes the stabilization of the oxygen-sensitive transcription factor hypoxia-inducible factor-1alpha (HIF-1α) that induces the expression of multiple genes implicated in angiogenesis, metabolism, and cell survival [1, 2]. Of the prominent gene products, vascular endothelial growth factor (VEGF) strongly stimulates the formation of new blood vessels (angiogenesis) and vascular networks that supply both nutrients and oxygen to tumor cells, resulting in tumor growth and invasion as well as metastasis [3, 4]. Thus, antiangiogenic treatment that targets the VEGF/VEGF receptor (VEGFR) pathway is a potential strategy to inhibit tumor vessel growth, thus abrogating tumor progression and metastasis.

Several studies have focused on the development of humanized monoclonal antibodies and small molecules that inhibit tumor angiogenesis by targeting the tumor-associated endothelial cells [5–7]. Angiogenesis inhibitors including neutralizing VEGF antibodies and receptor-tyrosine kinase inhibitors, such as bevacizumab, ramucirumab, sunitinib, and axitinib, have been developed and used clinically to treat several solid tumors including colorectal cancer and renal cell carcinoma [8]. Recently, they reduce tumor blood vessel abnormalities (that are, vascular junction destabilization, pericyte loss, and vascular permeability and leakage) and improve delivery of anticancer drugs to tumor, thereby potentiating efficacy of their antitumor activity [9, 10]. However, the anti-VEGF antibody bevacizumab has some limitations due to adverse effects of arterial hypertension, arterial and venous thrombosis, cardiovascular events, and gastrointestinal bleeding [11]. In addition, small chemical drugs that inhibit VEGF receptor tyrosine kinase can also suppress several other tyrosine kinase activities due to low specificity, thus resulting in unwanted side effects, such as hypertension or proteinuria [12, 13].

Specific inhibitors targeting VEGFR-2, a primary receptor for VEGF family members driving angiogenesis, are needed for inhibition of tumor angiogenesis and vessel leakage. Ramucirumab is a fully humanized monoclonal antibody bound to the extracellular domain of VEGFR-2. Ramucirumab treatment of various malignancies has shown promising clinical antitumor efficacy and tolerability. Although ramucirumab has been suggested to elicit hypertension as an adverse event [14], it can be manageable [15]. Thus, VEGFR-2 is a promising target for treatment of cancer patients.

Angiostatin, a potent inhibitor of angiogenesis, was first identified as an endogenous kringle (domains 1-4)-containing fragment produced from plasminogen, which selectively inhibits endothelial cell proliferation [16]. Thereafter, a single kringle 5 of plasminogen was shown to be more effective in anti-endothelial cell proliferation than angiostatin [17], and the tetrapeptide Lys-Leu-Tyr-Asp (KLYD) derived from the kringle 5 was revealed to have a potent inhibitory effect on endothelial cell proliferation [18]. We recently demonstrated that Arg-Leu-Tyr-Glu (RLYE) can more effectively inhibit VEGF-induced angiogenic activity *in vitro* than KLYD [19]. However, its underlying mechanism of action is unclear, as are the pharmacological effects on tumor angiogenesis, tumor blood vessel leakage, and tumor progression in an animal model.

In this study, we examined the therapeutic effects of RLYE on tumor progression and its molecular target for antiangiogenic therapy. RLYE inhibited VEGF-A-induced angiogenesis *in vivo* by directly binding to VEGFR-2, but not VEGFR-1, and subsequently blocked the interaction between VEGF-A and VEGFR-2 and its downstream signaling cascades. Thus, the peptide inhibited tumor growth and metastasis by suppressing tumor angiogenesis. In addition, RLYE potentiated the synergistic effect of the cytotoxic agent irinotecan on tumor cell apoptosis and tumor growth inhibition, probably by increasing drug delivery to tumor, via reduction of tumor vessel abnormality due to restoration of endothelial adherens junction and pericyte coverage. These findings demonstrate that RLYE is a potent antiangiogenic and vascular remodeling drug that binds to VEGFR-2, thus providing a new therapeutic strategy for solid tumors.

## RESULTS

### RLYE inhibits angiogenesis *ex vivo* and *in vivo*

**Figure 1:**
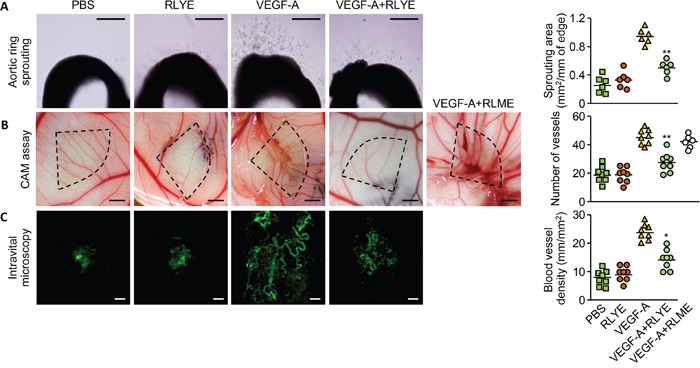
RLYE inhibits angiogenesis ex vivo and *in vivo* **A**. Rat aortic rings were incubated with VEGF-A, RLYE or combined together. On day 6, newly formed vessels were fixed and microvessel outgrowth was photographed under a phase contrast microscope. Scale bar, 200 μm. **B**. Thermanox discs containing VEGF-A, RLYE, VEGF-A plus RLYE or VEGF-A plus RLME were loaded onto the CAM of 10-day-old embryos. After 72 h incubation, the area around the loaded disc was photographed. Scale bar, 2 mm **C**. Matrigel containing VEGF-A alone or in combination with RLYE was applied to the inner space of window, which was surgically implanted between the skin and abdominal wall of mice. After 4 days, neovascularization was recorded using a fluorescence microscope after intravenous injection of FITC-labeled dextran. Angiogenesis was assessed as described in the Methods section. Scale bar, 200 μm. Data are the mean ± SD (n ≥ 6). * *P* < 0.05 and **P < 0.01 versus VEGF-A alone.

Since RLYE inhibits *in vitro* angiogenic behaviors, such as proliferation, migration, and tube-like structure formation, of HUVECs treated with VEGF-A [19], we hypothesize that RLYE can inhibit tumor growth and metastasis via inhibition of tumor angiogenesis. To confirm this hypothesis, we first examined the effects of RLYE on angiogenesis *ex vivo* and *in vivo*. In an *ex vivo* angiogenesis assay using explanted rat aortic rings in Matrigel matrices, RLYE significantly inhibited vessel sprouting in the cut edge of aortic rings exposed to VEGF-A (Figure 1A). In addition, similar results were also obtained in mouse aortic ring sprouting assay (Supplementary Figure 1). We also investigated whether RLYE is capable of regulating *in vivo* angiogenesis using the chick chorioallantoic membrane (CAM) assay. RLYE treatment markedly suppressed the total surface density of capillaries induced by VEGF-A (Figure 1B). However, the peptide RLME that has no antiangiogenic activity [19] did not inhibit VEGF-induced angiogenesis in the CAM model (Figure 1B). We further confirmed the antiangiogenic capability of RLYE in an animal model using intravital microscopy. Treatment with RLYE effectively blocked VEGF-A-induced increases in the angiogenic characteristics of capillary sprouting and neovessel formation (Figure 1C). These results indicate that RLYE is capable of inhibiting VEGF-A-induced neovessel formation *in vivo*.

### RLYE blocks VEGF-induced angiogenic signaling by inhibiting VEGFR-2 activation

**Figure 2:**
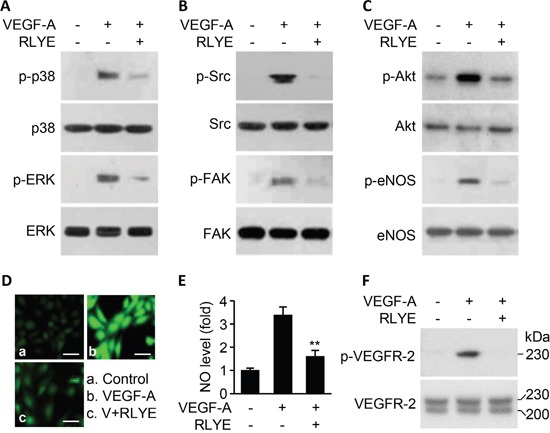
RLYE inhibits VEGF-A-induced angiogenic signal cascades HUVECs were treated with VEGF-A (10 ng/ml) alone or in combination with RLYE (0.15 nM) for 30 min, except for measurement of NO in cells that were incubated for 4 h. Cell lysates were separated by SDS-PAGE, followed by Western blotting to determine the phosphorylation levels of p38MAPK and ERK **A**. Src and FAK **B**. Akt and eNOS **C**. and VEGFR-2 (F). **D** and **E**. The levels of intracellular NO were determined by confocal microscopy using DAF-FM. Scale bar, 50 μm. **F**. Two VEGFR-2 bands with MW of 220 and 230 kDa indicate intermediate and mature forms, respectively. Data are the mean ± SD (n = 6). **P < 0.01 versus VEGF-A alone.

To understand the molecular mechanism by which RLYE inhibits VEGF-induced angiogenesis, we examined the effect of RLYE on intracellular signaling events triggered by VEGF-A. Treatment of HUVECs with RLYE inhibited several angiogenic signals, such as the cell proliferation signals p38 and ERK activation, the cell migration signals Src and FAK phosphorylation, and the cell survival signal Akt phosphorylation, in HUVECs stimulated with VEGF-A (Figure 2A-2C). In addition, RLYE effectively blocked VEGF-A-induced endothelial nitric oxide synthase (eNOS) phosphorylation and NO production (Figure 2C-2E), which improve endothelial and vascular function [20] Furthermore, RLYE inhibited the apical angiogenic signal event VEGFR-2 phosphorylation in HUVECs treated with VEGF-A (Figure 2F). These results suggest that RLYE inhibits VEGF-A-induced signal cascades by inhibiting VEGFR-2 phosphorylation.

### RLYE does not inhibit angiogenesis induced by basic fibroblast growth factor (bFGF), epidermal growth factor (EGF), and sphingosine 1-phosphate (S1P)

**Figure 3:**
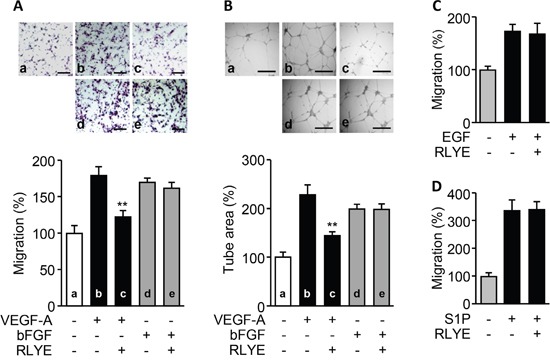
RLYE inhibits *in vitro* angiogenesis induced by VEGF-A, but not bFGF, EGF, and S1P HUVECs were incubated with VEGF-A (10 ng/ml), bFGF (10 ng/ml), EGF (20 ng/ml) or S1P (1 nM) in the presence or absence of RLYE (0.15 nM). **A**. Endothelial cell migration was determined by the Boyden chamber assay. Cells that migrated to the lower side of the filter were counted. Scale bar, 100 μm. **B**. Images of tube-like structure were photographed using an inverted phase contrast microscope, and the tube length was quantified using Image-Pro Plus software. Scale bar,500μm. **C** and **D**. Endothelial cell migration was determined by Boyden chamber assay. Data are the mean ± SD (n = 3). **P < 0.01 versus VEGF alone.

We next investigated whether RLYE inhibits angiogenesis induced by other angiogenic factors, such as bFGF, EGF and S1P. Treatment of RLYE did not inhibit bFGF-induced increases in human endothelial cell migration and tube formation, while this peptide effectively suppressed VEGF-A-induced angiogenesis (Figure 3A and 3B). In addition, RLYE did not inhibit EGF-induced endothelial cell migration (Figure 3C). Since the bioactive lipid S1P stimulates endothelial cells to promote angiogenesis [21], we next examined the regulatory effect of RLYE on S1P-induced angiogenesis. S1P strongly increased endothelial cell migration, and this effect was not inhibited by RLYE (Figure 3D). However, the peptide did not induce any cytotoxicity against HUVECs (Supplementary Figure 2A). These findings suggest that RLYE inhibits angiogenesis induced by VEGF-A, but not by other angiogenic factors including bFGF, EGF, and S1P.

### RLYE inhibits angiogenesis induced by VEGFR-2, but not VEGFR-1

**Figure 4:**
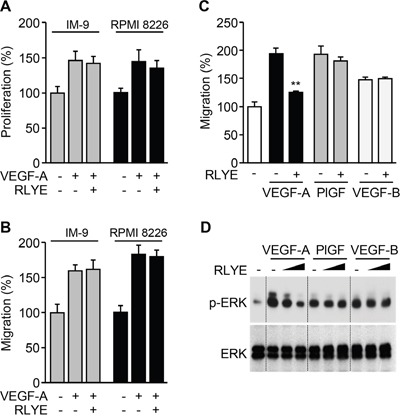
RLYE inhibits *in vitro* angiogenic events induced by VEGFR-2, but not VEGFR-1 **A**. and **B**. IM-9 and RPMI 8226 cells were incubated VEGF-A alone or in combination with RLYE. (A) Cell proliferation was determined by [^3^H]-thymidine incorporation assay. (B) Endothelial cell migration was determined by Boyden chamber assay. **C** and **D**. HUVECs were incubated with VEGF-A (10 ng/ml), PlGF (20 ng/ml) or VEGF-B (20 ng/ml) in the presence or absence of RLYE (0.15 nM). (C) Endothelial cell migration was determined in a Boyden chamber. (D) ERK phosphorylation was determined by Western blotting. Data are the means ± SD (n = 3). **P < 0.01 versus VEGF-A alone.

Since VEGF-A activates both VEGFR-1 and VEGFR-2 that are expressed in endothelial cells [22], we investigated whether RLYE inhibited one or both of these receptors. We first examined the effects of RLYE on proliferation and migration of two multiple myeloma (MM) cell lines, IM-9 and RPMI 8226 cells, which express VEGFR-1, but not VEGFR-2 [23, 24]. Interestingly, RLYE did not suppress VEGF-A-induced proliferation and migration of both cell lines (Figure 4A and 4B). We next examined the effect of RLYE on migration of HUVECs stimulated with VEGF-A (ligand for VEGFR-1/2), placental growth factor (PlGF, a ligand for VEGFR-1), and VEGF-B (ligand for VEGFR-1). RLYE markedly inhibited endothelial cell migration elicited by VEGF-A, but not PlGF and VEGF-B (Figure 4C). As expected, similar inhibitory effects of RLYE on the angiogenic signal ERK activation were observed in HUVECs stimulated with VEGFR-1 and VEGFR-2 ligands (Figure 4D). These results suggest that RLYE inhibits angiogenic phenomena induced by VEGFR-2, but not VEGFR-1.

### RLYE interacts with VEGFR-2, but not VEGFR-1

**Figure 5:**
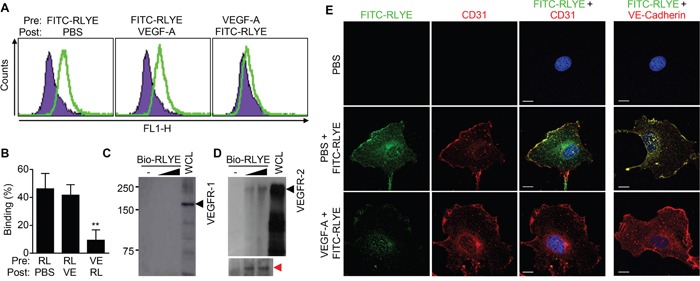
RLYE binds to endothelial cells through binding to VEGFR-2 **A** and **B**. HUVECs were incubated with FITC-conjugated RLYE (RL, 15 nM) before or after treatment with VEGF-A (VE, 100 ng) for 30 min. Cells were analyzed by flow cytometry (A) and quantified (B). Data are the means ± SD (n = 5). **P < 0.01 versus RLYE alone. **C** and **D**. HUVEC lysates were incubated with biotinylated RLYE (Bio-RLYE, 0.15 or 1.5 nM), followed by precipitation with streptavidin-agarose beads. The red arrow indicates VEGFR-2 protein bands on a longer exposure film. The precipitates were separated by SDS-PAGE. VEGFR-2 or VEGFR-1 expression was assessed by Western blot analysis. **E**. HUVECs were treated with or without VEGF-A (10 ng/ml) for 30 min, followed by incubation with FITC-conjugated RLYE (100 ng/ml, green). Cells were incubated with a TRITC-conjugated CD31 antibody (1:100, red) or DAPI (blue). Confocal microscopy was performed for analyzing binding of RLYE on HUVECs. Scale bar, 10 μm.

We next investigated whether RLYE binds to VEGFR-2 in endothelial cells. HUVECs were incubated with FITC-conjugated RLYE before or after VEGF-A treatment, and FACS was performed to determine the binding capacity of RLYE to endothelial cells. RLYE bound to the surface of control HUVECs, and this binding was blocked by pre-, but not post-, incubation of endothelial cells with VEGF-A (Figure 5A and 5B). In addition, RLYE binding was colocalized with the endothelial cell marker CD31 (PECAM-1) or VE-cadherin. Moreover, a pull-down assay showed that RLYE bound to VEGFR-2, but not VEGFR-1, expressed in HUVECs (Figure 5C and 5D). Under similar experimental condition, confocal analysis showed that RLYE bound to the surface of HUVECs, and this binding was effectively blocked by pretreatment with VEGF-A (Figure 5E). Since the surface VEGFR-2 of endothelial cells undergoes internalization (to the nucleus) by VEGF ligation [25], we further examined whether RLYE inhibits nuclear translocation of VEGFR-2 in HUVECs stimulated with VEGF-A. VEGF-A treatment increased nuclear level of VEGFR-2, accompanied by a decrease in membrane and cytosolic VEGFR-2 level, while RLYE did not affect VEGF localization (Supplementary Figure 3). Interestingly, pretreatment with RLYE impaired VEGF-A-induced internalization of VEGFR-1, and RLYE post-treatment inhibited partially VEGF-A-induced nuclear localization of VEGFR-2 (Supplementary Figure 3). These results suggest that RLYE impaired VEGFR-2 internalization by binding to the receptor and then blocking VEGF-A ligation.

### An *in silico* modeling of RLYE binding to VEGFR-2

**Figure 6:**
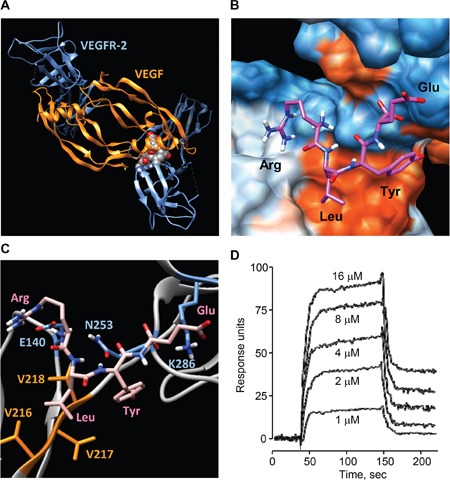
Binding of RLYE to VEGFR-2 **A**. *In silico* molecular docking structure of the RLYE peptide (red spheres) bound at the interface between VEGFR-2 (blue) and VEGF (orange). **B**. Binding model of RLYE to VEGFR-2. Red and blue colors represent hydrophobic and hydrophilic regions, respectively. **C**. Interaction of the RLYE peptide (pink) with VEGFR-2. The residues E140, N253, and K286 (blue) of VEGFR-2 are involved in electrostatic interaction and hydrogen bonding with Arg and Glu of the RLYE peptide whereas Val residues (orange) participate in hydrophobic interaction Leu of the peptide. **D**. SPR analysis was performed for determining the interaction between RLYE and rhVEGFR-2.

To investigate the possible interaction of RLYE with VEGFR-2, a docking analysis was performed. Blind docking of RLYE to VEGFR-2 produced a tightly bound conformation with a small dissociation constant (194 nM). Interestingly, RLYE bound to VEGFR-2 at the interface between immunoglobulinhomology domains D2 and D3 (Figure 6A), which is known to be the binding site for VEGF-A/C [26]. Charged residues at both termini of the peptide bind to hydrophilic pockets, while other non-charged residues were placed close to hydrophobic patch (Figure 6B). The terminal Arg and Glu residues of the peptide bind electrostatically to Glu140 and Lys286 of VEGFR-2, respectively, whose Lys286 is known to form a salt bridge with Glu64 of VEGF-A (Figure 6C) [26]. The Arg residue also formed a hydrogen bond with Asn253 of VEGFR-2, which is critically involved in interaction with VEGF-A [26]. The Leu side chain contacted a hydrophobic region comprised of Val216-218 of VEGFR-2, which elicit a hydrophobic interaction with VEGF-A [26], thus favoring hydrophobic interaction between RLYE and VEGFR-2. On the other hand, the aromatic ring of Tyr also interacted hydrophobically with Val216-218 residues of VEGFR-2, while its hydroxyl group formed a hydrogen bond with the amide carbonyl of Asn253. These results suggest that RLYE binds to VEGFR-2 at the same binding site of VEGF-A. Next, the binding between RLYE and rhVEGFR-2 was demonstrated by SPR analysis. The sensorgrams displayed saturable binding behavior of RLYE to rhVEGFR-2, and Kd of RLYE binding to VEGFR-2 was calculated as 9.0 μM (Figure 6D). These results suggest that RLYE sterically blocks the ligand VEGF-A binding by interaction with VEGFR-2 and potentially inhibits VEGF-A-induced angiogenesis.

### RLYE inhibits growth and metastasis of mouse melanoma tumors in a mouse model

**Figure 7:**
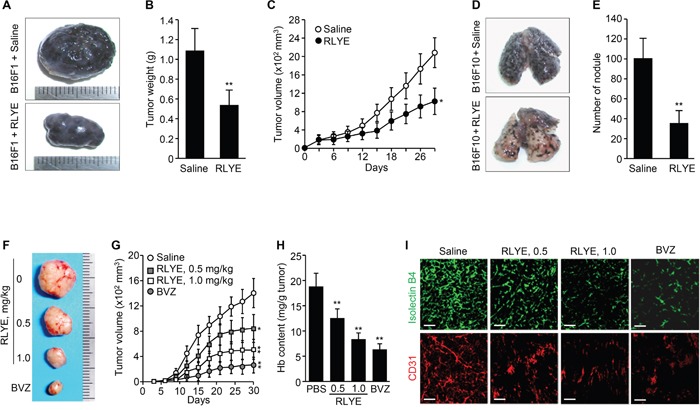
RLYE inhibits tumor growth and metastasis by suppressing tumor angiogenesis **A**. Representative tumors isolated from 29-days-old B16F1 tumor-bearing C57BL/6J mice injected i.p. with saline or RLYE (1 mg/kg) every day. **B**. Tumor weight on day 29 after injection of B16F1 cells (n = 10 mice per group). **C**. Time course inhibition of B16F1 tumor growth by treatment with RLYE (n = 10 mice per group). **D**. Representative images for metastatic nodules of the lungs isolated from C57BL/6J mice, which were injected i.v. with B16F10 melanoma cells and treated with saline or RLYE for 3 weeks. **E**. Quantification of the number of lung metastatic nodules. **F-I**. HCT116 tumor-bearing nude mice treated with saline, RLYE (0.5 mg/Kg or 1.0 mg/Kg) every day or bevacizumab (BVZ, 2 mg/kg) twice a week. Representative tumors from 30-day-old HCT116 tumor-bearing mice. (G) Tumor burden was measured every 3 days (n = 8 mice per group). (H) Functional vascularization was quantified by measuring hemoglobin concentration in HCT116 tumor tissues (n = 5). (I) Representative image of tumor sections that were stained with isolectin B4 and an anti-CD31 antibody. Scale bar, 100 μm. Data are the mean ± SD. * *P* < 0.05 and **P < 0.01 versus saline treatment.

Since VEGFR-2 is critically involved in tumor angiogenesis [18], we next evaluated the pharmacological effects of RLYE on tumor progression in a mouse model. B16F1 melanoma cells were injected s.c. into the flanks of mice, and tumor-bearing mice were injected i.p. with RLYE at a dose of 1 mg/kg/day. Treatment with RLYE resulted in a significant decrease of tumor size, weight, and growth (Figure 7A-7C). To explore the inhibitory effect of RLYE on tumor metastasis *in vivo*, mice were injected with B16F10 melanoma cells into the tail vein and injected i,p. with RLYE at a dose of 1 mg/kg/day. Three weeks later, metastatic colonies were analyzed in the lungs. Administration with RLYE resulted in a 62% reduction in number of metastatic colonies in the lungs compared with untreated control mice (Figure 7D and 7E). These results suggest that RLYE is capable of inhibiting tumor growth and metastasis.

### RLYE inhibits human xenograft tumor growth via antiangiogenic action

To investigate the effect of RLYE on human tumor growth and tumor angiogenesis in a xenograft mouse model, nude mice bearing subcutaneous human colorectal tumor (HCT116) xenograft were injected i.p. with 0.5 or 1.0 mg/kg every day. Treatment with RLYE significantly decreased the tumor size and inhibited tumor growth in a concentration-dependent manner (Figure 7F and 7G), while the peptide showed no cytotoxic effect on cultured HCT116 cells up to 30 nM, which is relatively equal to the blood concentration of mice (22 g of body weight) injected with 1 mg/kg (Supplementary Figure 2B). Hemoglobin concentration in tumor tissues was determined to assess functional angiogenesis [27]. RLYE-treated tumors had a reduction in 41% hemoglobin content compared to control tumors (Figure 7H). Immunohistochemistry of tumor tissues showed that RLYE treatment resulted in dose-dependent decrease in staining with FITC-isolectin B4 and an anti-CD31 (PECAM-1) antibody (Figure 7I), indicating that the peptide inhibits tumor angiogenesis. The inhibitory effects of RLYE on tumor growth and angiogenesis were relatively little lower than those of bevacizumab (2 mg/kg, twice per week) as a positive control. These results suggest that the antitumor efficacy of RLYE is closely correlated with its antiangiogenic effect in a xenograft mouse model.

### RLYE inhibits vascular leakage and enhances tumor chemosensitivity

**Figure 8:**
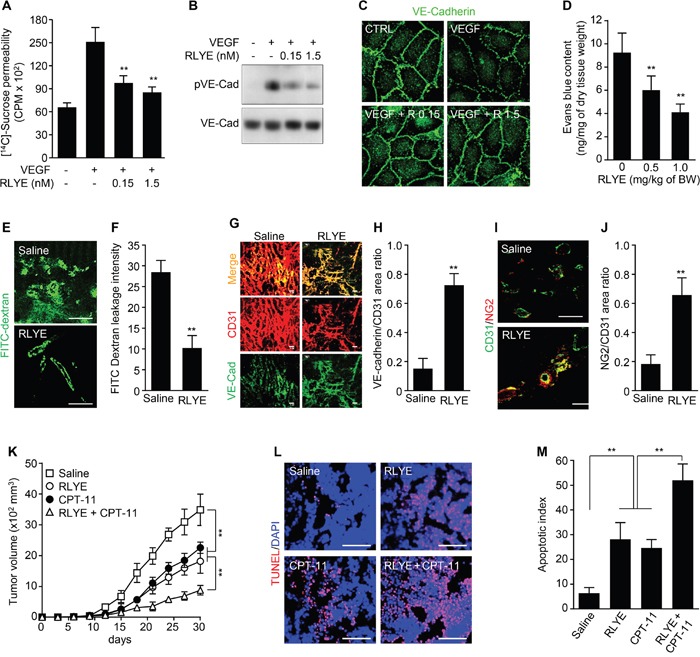
RLYE inhibits vascular leakage and enhances tumor chemosensitivity **A**. Endothelial cell permeability was determined by diffusion of [^14^C]-sucrose through HUVEC monolayers treated with 20 ng/ml of VEGF alone or in combination with RLYE (n = 3). **B**. Phosphorylation of VE-cadherin was determined in cell lysates of HUVECs treated with 20 ng/ml of VEGF alone or in combination with RLYE. **C**. Immunostaining of VE-cadherin in endothelial monolayer was determined by confocal microscopy. **D-J**. HCT116 tumor-bearing mice (n = 5 per group) were injected with saline or RLYE (0.5 mg/kg) for 12 days. Tumor vascular leakage was determined by the Evans blue method (E and F) or FITC-dextran image assay (G and H). (G) Immunofluorescence staining of the tumor sections (n = 5) for CD31 and VE-cadherin, and (H) ratio of VE-cadherin to CD31 was quantified using computer-aided confocal microscopy. (I) Immunofluorescence staining of the tumor sections (n = 5) for CD31 and NG2, and (J) NG2-positive vessels were quantified. **K-M**. HCT116 tumor-bearing mice (n = 7 per group) were i.p. injected with RLYE (0.5 mg/kg/day) alone or in combination with irinotecan (CPT-11, 17 mg/kg every 5^th^ day). (K) Tumor volumes were measured every 3 days. (L and M) Apoptotic cells in tumor tissues were determined and quantified by TUNEL staining. Scale bar, 100 μm in all images. **P<0.001 (Student's *t*-test except survival curve data).

VEGF has a prominent role in leakiness of tumor blood vessels, hence, we first investigated the effect of RLYE on VEGF-induced endothelial cell permeability in cultured HUVECs. RLYE significantly attenuated VEGF-induced sucrose permeability in endothelial cell monolayers (Figure 8A). RLYE also inhibited VEGF-induced phosphorylation of VE-cadherin, an adherens junction protein, resulting in prevention of VEGF-mediated VE-cadherin loss from endothelial adherent junctions (Figure 8B and 8C). The results suggest that RLYE attenuates VEGF-induced endothelial permeability by blocking VEGFR-2. In addition, the Evans blue method in HCT116 tumor-bearing nude mice demonstrated that RLYE reduced tumor blood vessels leakage (Figure 8D). Similar effect of RLYE on blood vessel leakage was observed in the human tumor xenograft model by FITC-dextran image assay (Figure 8E and 8F). These data suggest that RLYE inhibits tumor vessel hyperpermeability, which is largely regulated by endothelial cell junction and pericyte coverage [9]. We next examined the effect of RLYE on endothelial cell-cell junction and pericyte coverage of tumor vessels, an indicator of vascular stabilization and functionality. RLYE increased the numbers of VE-cadherin-positive vessels (Figure 8G and 8H) and the pericyte marker NG2-positive vessels in HCT116 tumors (Figure 8I and 8J). Reducing vascular leakage enhances drug delivery to tumor and chemotherapeutic efficacy [9, 10]. Therefore, we evaluated the therapeutic effect of co-treatment with RLYE and the anticancer drug irinotecan (camptothecin-II, CPT-11) on tumor progression in a mouse model. Treatment with the peptide or CPT-11 alone significantly inhibited tumor growth in mice bearing HCT116 colon tumor. The growth inhibition was further augmented in the group treated with combination of RLYE and CPT-11 (Figure 8K). TUNEL-positive apoptotic cells were markedly increased in tumor tissues of the combination treatment group, as compared with the monotherapy groups (Figure 8L and 8M). These results suggest that RLYE reduces tumor blood vessel permeability and leakage via promotion of endothelial junction stability and pericyte coverage, leading to enhanced anticancer drug delivery to tumor and therapeutic efficacy.

## DISCUSSION

Since angiogenesis is necessary for tumor progression and metastasis, angiogenesis inhibitors are a clinically validated anticancer drug. In the present study, we examined the antiangiogenic mechanism of the tetrapeptide RLYE and its therapeutic effect on tumor growth and metastasis. The peptide suppressed angiogenic activity of VEGF-A, but not of VEGF-B, bFGF, PlGF, and S1P, by directly binding to VEGFR-2, but not VEGFR-1, in human endothelial cells. Furthermore, RLYE showed antitumor activity in both C57BL/6J mice bearing melanoma B16F10 tumors and athymic nude mice bearing colorectal HCT116 tumors, as well as suppressed lung metastasis of B16F1 melanoma in a mouse model. This peptide also inhibited tumor angiogenesis in a mouse xenograft model of colorectal cancer. These findings suggest that RLYE possesses strong therapeutic potential for solid tumors via antiangiogenic action.

Hypoxic tumor microenvironment elicits stabilization of HIF-1α and subsequent induction of VEGF expression. VEGF increases tumor angiogenesis and vascular networks that supply both nutrients and oxygen to tumor cells, resulting in increases in tumor growth and metastasis [3, 4]. The VEGF family is comprised of the five VEGF isoforms (VEGF-A, VEGF-B, VEGF-C, VEGF-D, and VEGF-E) and PlGF and binds to three primary receptors [22]. Although VEGFR-1 and VEGFR-2 are expressed mainly in endothelial cells and associated with angiogenesis, they have distinct biological functions. VEGFR-1 has high binding affinity for VEGF-A, VEGF-B, and PlGF, but weak angiogenic activity, as compared with VEGFR-2, suggesting a negative inhibitory role in VEGFR-2-induced angiogenesis [28, 29]. However, VEGFR-2 exhibits low binding affinity to VEGFs, but high activity of angiogenic signaling, indicating that VEGFR-2 is a main receptor driving angiogenesis under physiological and pathological conditions.

Based on the biological properties of VEGFR-1, the soluble decoy VEGFR-1 aflibercept that has several hundred-fold greater affinity to VEGF-A than bevacizumab has been developed as an antiangiogenic drug for the treatment of colorectal cancer [30, 31]. Therefore, the VEGF-A/VEGFR-2 system is widely considered a main target of the therapeutic treatment for tumor angiogenesis. Ligation of VEGFR-2 triggers strong proangiogenic signals including p38MAPK/ERK, Src/FAK, and Akt/eNOS through VEGFR-2 phosphorylation, which promote angiogenesis and VEGFR-2 internalization. Our data show that RLYE blocked the angiogenic signals, angiogenesis, and VEGFR-2 internalization induced by VEGF-A. This suggests that RLYE is an inhibitor of VEGF-A/VEGFR-2 signaling pathways.

Successful blockade of the VEGFR-2 pathway is a potential therapeutic strategy to inhibit tumor angiogenesis and tumor progression. Disruption of VEGFR-2 signaling occurs through three different modes of action by antiangiogenic drugs: specific binding to circulating VEGFs, interference with VEGFs/VEGFR2 interaction, and inhibition of VEGFR-2-tyrosine kinase [32]. Although several VEGFR-2-tyrosine kinase inhibitors have been clinically used, there are some therapeutic limitations, such as development of resistance, lack of tumor response in the general population, and low specificity [13, 33]. Humanized anti-VEGF antibodies including bevacizumab have been integrated into the treatment of patients with different types of cancers. However, recent studies reported that cancer patients with VEGF gene polymorphisms do not respond very well to bevacizumab, and the development of bevacizumab-resistant tumors has become more common [34, 35]. Therefore, other therapeutic approaches including the use of humanized anti-VEGFR-2 antibody ramucirumab to block VEGFR-2 have been more attractive to inhibit tumor angiogenesis [36, 37]. Similarly, our data demonstrate that the tetrapeptide RLYE inhibits VEGF-A-induced tumor angiogenesis by directly binding to VEGFR-2.

RLYE has been shown to inhibit the angiogenic signal cascades in HUVECs stimulated with VEGF-A at a maximum inhibitory molar ratio of 11:1 of RLYE to monomeric VEGF [19], which is different from the approximately 1:1 molar ratio of bevacizumab [38]. This suggests that the peptide does not directly bind to VEGF-A, which is different from the mode of action of bevacizumab. However, our results show that RLYE inhibits the VEGF-A-induced signal pathway by interfering with specific binding of VEGF-A to VEGFR-2. This evidence was further confirmed by the findings that RLYE inhibited *in vitro* angiogenesis in HUVECs stimulated with VEGF-A, but not with VEGFR-1-specific ligands (PlGF and VEGF-B), and that the peptide did not inhibit VEGF-A-induced proliferation and migration of MM cell lines that expressed only VEGFR-1. These results suggest that RLYE inhibits the VEGF-A/VEGFR-2 pathway, but not the VEFG-A/VEGFR-1 system. This is further supported by biochemical and immunochemical experiments using pull-down assay, FACS, and SPR analysis. Interestingly, our modeling study predicted that this peptide bound to VEGFR-2 at the same site that interacts with VEGF-A [26, 39–41]. These results suggest that RLYE inhibits angiogenic activity of VEGF-A, a major isoform of VEGF family, by binding to VEGFR-2 and subsequently blocking VEGF-A/VEGFR-2 interaction.

Our previous study showed that RLYE has potent antiangiogenic activity with IC_50_ of 0.06-0.08 nM against VEGF-A [19]. This activity is more potent than those of clinically used antiangiogenic cancer drugs including bevacizumab (IC_50_ = 0.15 nM for VEGF-A), ramucirumab (IC_50_ = 1-2 nM for VEGFR-2), and the tyrosine receptor kinase inhibitor sunitinib (IC_50_ = 80 nM for VEGFR-2) in endothelial cell culture conditions [36, 38, 42]. However, we found the antiangiogenic and antitumor activities of RLYE were relatively little lower than those of bevacizumab in a xenograft mouse model. This unexpected results may be due to a short half-life (1.2 h) of RLYE in blood, compared with its half-life of >18 h in 10% heat-inactivated FBS-supplemented M199 media (unpublished data) and a half-life (12-22 days) of bevacizumab *in vivo* [43]. These results support that RLYE effectively inhibits tumor progression likely by blocking tumor angiogenesis, although it has a short half-life *in vivo*. We are now developing stable peptides with a long half-life *in vivo*, based on amino acid sequence of RLYE, which are used to effectively inhibit VEGFR-2 function in vascular endothelial cells and thereby inhibit tumor angiogenesis, upon which solid tumors depend for growth and metastasis.

Blockade of the VEGF-A signaling pathway inhibits tumor angiogenesis and transiently improves tumor vessel normalization and perivascular cell coverage, leading to reduced vessel permeability and increased vascular perfusion in tumors [9, 10]. Tumor vessel normalization improves delivery of cancer drugs and therapeutic efficacy. The antiangiogenic antibody bevacizumab with the anticancer drug topotecan significantly inhibits tumor growth, compared with each monotherapy, via tumor vessel normalization in a human brain tumor xenograft model [44]. Similarly, in an earlier study, we show that the vascular leakage blocker Sac-1004 potentiates cisplatin-induced antitumor activity via restoration of vascular normalization in tumor-bearing mice [45]. The present data demonstrated that RLYE inhibits tumor vessel permeability and leakage by restoring endothelial junction formation and pericyte coverage, thereby potentiating irinotecan-mediated tumor cell apoptosis and tumor growth inhibition in the tumor-bearing mouse model. Thus, RLYE reduces tumor vessel abnormality and permeability, resulting in enhanced delivery and efficacy of chemotherapeutic agents.

In conclusion, the present study supported the role of RLYE in the prevention of tumor progression and metastasis by selectively inhibiting functional neovessel formation within rapidly growing solid tumors in a mouse model. RLYE also potentiate antitumor activity of cytotoxic anticancer drugs by improving tumor vessel normalization via reduction of vascular permeability and leakage. Moreover, its specific inhibitory effect on the VEGFR-2-mediated signaling pathway could be targeted for the development of pharmaceutical agents that inhibit tumor angiogenesis and tumor blood vessel leakage. This proof of concept study provides the rationale for further investigation of RLYE as an antitumor agent, antiangiogenic drug or vascular leakage blocker in clinically advanced or metastatic solid tumors.

## MATERIALS AND METHODS

### Materials

Cell culture media and supplements were purchased from Invitrogen Life Technologies (Carlsbad, CA). Fetal bovine serum (FBS) was obtained from HyClone Laboratories (Logan, UT), and human bFGF and S1P were from Upstate Biotechnology (Lake Placid, NY). Human recombinant proteins including VEGF-A_165_, VEGF-B, EGF, PlGF, and VEGF receptor-2 (rhVEGFR-2) were obtained from R&D Systems (Minneapolis, MN). RLYE, fluorescein isothiocynate (FITC)-conjugated RLYE, and biotin-labeled RLYE were purchased from Peptron (Daejeon, South Korea). All peptides were dissolved in phosphate-buffered saline (PBS) or saline at a concentration of 15 mM as a stock solution. Antibodies for phospho-ERK (Thr-202/Tyr-204), phospho-Akt (Ser-473), phospho-p38 (Thr-180/Tyr-182), phospho-Src (Tyr-416), phosphor-VEGFR-2 (Tyr-1175), phospho-FAK (Tyr-925), ERK, FAK, VEGFR-2, and Akt were obtained from Cell Signaling Technology (Beverly, MA). Antibodies for phospho-eNOS (Ser-1177) and eNOS were purchased from BD Transduction Laboratories (San Diego, CA). Antibodies for VEGFR-1 and p38 were purchased from Santa Cruz Biotechnology (Santa Cruz, CA). Bevacizumab was purchased from Roche (Basel, Switzerland). Thermanox disc was purchased from Nalge Nunc International (Naperville, IL).

### Cell culture

Mouse melanoma B16F1 and B16F10 cells and human HCT116 colon cancer cells were obtained from American Type Culture Collection (Manassas, VA) and cultured in RPMI medium supplemented with 10% FBS, 1 mM sodium pyruvate, 10 mM 4-(2-hydroxyethyl)-1-piperazineethanesulfonic acid, and 100 U/ml penicillin-streptomycin in a humidified atmosphere of 5% CO_2_ at 37°C. Human umbilical vein endothelial cells (HUVECs) were maintained and cultured in M199 as described previously [19], and only passages 2-7 were used for all experiments. Human multiple myeloma IM-9 and RPMI 8226 cells were obtained from Korean Cell Line Bank (Seoul, Korea). The cells were cultured in RPMI-1640 medium in a humidified CO_2_ incubator. Cell viability or cytotoxicity was evaluated by 3-[4,5-cimethylthiazol-2-yl]-2,5-diphenyl tetrazolium bromide (MTT, Sigma-Aldrich) assay.

### Animals

Seven-week-old male C57BL/6J or athymic nude mice and Sprague-Dawley rats were purchased from OrientBio (Seongnam, South Korea) and maintained on a standard (normal) chow diet *ad libitum* in a laminar airflow cabinet under specific pathogen-free conditions. Animal experiments were performed in accordance with the guidelines of the Institutional Animal Care and Use Ethics Committee of Kangwon National University. Moreover, this investigation conformed to the Guide for the Care and Use of Laboratory Animals published by the United States National Institutes of Health (NIH Publication, 8th Edition, 2011).

### *In vitro* angiogenesis assay

Angiogenic activity was determined by measurements of cell proliferation, migration, and tube formation as described previously [19]. HUVECs were pretreated with RLYE (0.15 nM) for 30 min and stimulated with several proangiogenic factors including VEGF-A (10 ng/ml), VEGF-B (20 ng/ml), bFGF (10 ng/ml), EGF (20 ng/ml), PlGF (20 ng/ml), and S1P (1 nM). Cell proliferation was determined by [^3^H]-thymidine incorporation assay. Chemotactic migration was analyzed using Transwell plates with 6.5-mm diameter polycarbonate filters (8 μm pore size). Tube-like structure formation was determined on a layer of growth factor-reduced Matrigel by an inverted phase-contrast microscope (×40) and quantified using the Image-Pro Plus version 4.5 (Media Cybernetics, San Diego, CA).

### *Ex vivo* and *in vivo* angiogenesis assay

Aortic ring sprouting assay was performed by a modified method based on a previous report [46]. Sprague-Dawley rats (6-week-old, male) and C57BL/6J mice (7-week-old, male) were anesthetized with inhaled halothane (5%) and then humanely sacrificed. Rat dorsal and mouse thoracic aortas were isolated and carefully cut into 1-mm rings. The aortic rings were placed in the 48-well plates pre-coated with 120 μl of Matrigel, sealed in place with an overlay of 50 μl of Matrigel, and incubated with RLYE (0.3 nM) or VEGF (20 ng/ml) in a final volume of 200 μl of serum-free medium. On day 6, newly formed vessels were fixed and microvessel outgrowth was photographed under a phase contrast microscope, and angiogenesis was quantified with Image J software (NIH; http://rsb.info.nih.gov/ij). For chick chorioallantoic membrane (CAM) assay, fertilized chick embryos were incubated for 3 days and then windowed as described previously [46]. Briefly, a window approximately 3 cm in diameter was formed by removing the shell and inner shell membrane from the air space site and then the exposed area was sealed with cellophane tape. The eggs were returned to the incubator at 37°C (humidity 55-60%) and incubated with the window upright for 3 days. On day 10, Thermanox discs containing 10 μl of salt-free solution containing RLYE (0.75 nM) alone or plus VEGF (50 ng/ml) were loaded onto the CAM of 10-day-old embryos. After 72 h incubation, the area around the loaded disk was photographed with a Nikon digital camera and the number of newly formed vessels was counted inside the disc area. Neovascularization was determined by intravital fluorescence microscopy as described previously [46]. C57BL/6Jmice were anesthetized by inhalation of 1.5% isoflurane and O_2_-N_2_O using a Surgivet vaporizer (Waukesha, WI), and titanium-based imaging windows were surgically implanted between the skin and abdominal wall of mice. Growth factor-reduced Matrigel (100 μl) containing RLYE (1.5 nM) or VEGF (100 ng) was applied to the inner space of the window, which was surgically implanted between the skin and abdominal wall of mice. After 4 days, neovascularization was recorded by a Zeiss Axiovert 200M microscope (Carl Zeiss, Jena, Germany) after intravenous injection of 50 μl of 25 mg/ml FITC-labeled dextran (MW, 250 kDa) via the tail vein. Vascular length density was calculated as the length of FITC-labeled dextran-perfused blood vessels per observation area (mm/mm^2^).

### Western blot analysis

Whole cell lysates were prepared using RIPA buffer. To prepare the membrane/cytosolic (M/C) and nuclear (N) fractions, cells were lysed into buffer A (10 mM HEPES, pH 7.9, 0.1 mM EDTA, 10 mM KCl, 0.1 mM EGTA and 0.1% Nonidet P-40) and centrifuged at 19,000 x *g* for 5 min. The *supernatant was used as an M/C fraction, and the pellet was* resuspended in buffer B (20 mM HEPES, pH 7.9, 0.4 M NaCl, 1 mM EDTA and 0.1 mM EGTA) and lysed by three cycles of freezing and thawing. After centrifugation at 19,000 x *g* for 5 min, the supernatant was isolated and used as a nuclear faction. Cell fractions (50 μg protein) were separated by SDS-PAGE and transferred to polyvinylidene difluoride membranes. The membranes were incubated with antibodies against target proteins for 2 h. After washing twice, the membranes were incubated with a horseradish peroxidase-conjugated secondary antibody, and protein levels were detected by an enhanced chemiluminescence system as described previously [46].

### Nitric oxide (NO) measurement

Intracellular NO levels were measured *in situ* using DAF-FM diacetate according to the manufacturer's instructions. HUVECs were pretreated with 0.15 nM peptide and stimulated with 10 ng/ml VEGF for 4 h. Cells were incubated with 5 μM (final concentration) 4-amino-5-methylamino-2',7'-difluorofluorescein (DAF-FM) diacetate for 30 min in a CO_2_ incubator. Intracellular NO levels were determined using a confocal laser microscope as described previously [19].

### Fluorescence-activated cell sorting (FACS) analysis

HUVECs cultured for 24 h after plating in 60 mm-culture dishes. Cells from subconfluent cultures were gently detached from wells with PBS containing 5 mM EDTA, washed three times with PBS, and resuspended in PBS containing 2% FBS/0.1% bovine serum albumin. Thereafter, they were incubated with FITC-conjugated RLYE (15 nM) before or after treatment with VEGF (100 ng/ml) for 30 min on ice, fixed in 2% paraformaldehyde, and analyzed by flow cytometry in a fluorescence-activated cell sorter (FACSCalibur, Becton Dickinson, Franklin Lakes, NJ).

### Pull-down assay of VEGFR using biotin-labeled RLYE

HUVECs were incubated with were biotinylated RLYE (0.15 or 1.5 nM) for 1 h, and the unbound peptide was washed away. Cells were solubilized in pull-down buffer (50 mM Tris-HCl, pH 8.0, with 150 mM sodium chloride, 1.0% NP-40, 0.5% sodium deoxycholate and 0.1% SDS). Cell lysates (500 μg protein) were incubated with streptavidin-agarose beads (20 μl) at 4°C for 1 h. RLYE-bound proteins were isolated form cell lysates by centrifugation, and the precipitated beads were boiled for 10 min. Proteins bound to the beads were separated by SDS-PAGE and probed by immunoblotting with an antibody against VEGFR-2 or VEGFR-1.

### Animal models of mouse melanoma and human colon cancer

C57BL/6J and nude mice were challenged subcutaneously (s.c.) in the left flank with 2 × 10^6^ B16F1 mouse melanoma cells and 1 × 10^7^ HCT116 human colon carcinoma cells in a volume of 100 μl, respectively. After the tumor volume became at least 50-70 mm^3^, which occurred within 7 days, the mice were injected intraperitoneally (i.p.) with saline, RLYE (0.5 or 1.0 mg/kg) every day or bevacizumab (2 mg/kg) twice a week from 6 to 30 days after tumor cell injection. Some mice were treated with RLYE (0.5 mg/kg/day) or in combination with irinotecan (CPT-11, 17 mg/kg) every fifth day. Tumor size was measured in two dimensions using calipers. Tumor volume (mm^3^) was calculated using the formula: width^2^ x length x 0.52 [16]. Tumor tissue was homogenized with PBS and centrifuged (12,000 x *g*, 5 min). The supernatant with hemoglobin was taken for analysis with Drabkin's reagent (Sigma-Aldrich). The level of hemoglobin derivative cyanmethemoglobin was measured by spectrophotometry, and the amounts of hemoglobin were calculated per 1 g of tumor.

### Experimental tumor metastasis model

C57BL/6J mice were injected with 1 × 10^5^ B16F10 mouse melanoma cells via the lateral tail vein. Mice were injected i.p. every day with 1 mg/kg RLYE in a volume of 100 μl for 3 weeks. The lungs were excised, rinsed in Dulbecco's PBS, and fixed overnight in 4% paraformaldehyde solution. The number of metastatic nodules present over the entire surface of the lungs was counted under a dissecting microscope.

### Endothelial cell and tumor vessel permeability

HUVECs were plated on Transwell plates and grown to formation of a confluent monolayer. Cells were incubated with M199 containing 1% FBS for 3 h and treated with various concentrations of RLYE for 30 min, followed by stimulation with 20 ng/ml of VEGF for 1 h. Fifty μl of [^14^C]-sucrose (0.8 μCi/ml; Amersham Pharmacia) was added to the upper compartment. After 30 min, endothelial cell permeability was determined by measuring the amount of radioactivity that diffused into the lower compartment using a liquid scintillation counter. Tumor vessel permeability was assessed by Evans blue dye and FITC-dextran as previously described [45]. Evans blue (50 mg/kg) was injected intravenously into HCT116 tumor-bearing mice treated with saline or RLYE (0.5 mg/kg/day) for 12 days, and tumors were excised 30 min later. They were dried at 60°C for 16 h and then dye was extracted with 1 ml formamide at 55°C for 16 h. The amount of extravasated Evans blue was determined by measuring absorbance at 620 nm using its standard solution by spectrophotometry. Vascular leakage visualization with FITC-dextran was achieved by an intravenous injection of 3 mg/mouse FITC-dextran (40-kDa; Sigma Aldrich) 10 min before capture of tumor. Tumors were then fixed briefly in 4% paraformaldehyde and cryosections were made to observe vascular leakage under fluorescence microscope.

### Surface plasmon resonance (SPR) assay

Binding kinetics and affinities of RLYE to rhVEGFR-2 were assessed using a BIAcore AB (Uppsala, Sweden). Carboxymethylated dextran biosensor chip (CM5, BIAcore AB) were activated with 1-ethyl-3-(3-dimethylaminopropyl and *N*-hydroxysuccinimide according to the supplier's instructions. Successful immobilization of rhVEGFR-2 was achieved by injecting rhVEGFR-2 (1 ng/μl in HBS-EP buffer containing 10 mM Hepes, 150 mM NaCl, 3 mM EDTA, and 0.005% Tween-20) onto the activated CM5 chip. To obtain kinetic data, different concentrations of RLYE in HBS-EP buffer were injected over the sensor chip at a flow rate of 25 μl/min, and the association and dissociation behavior were compared. Peptide binding was measured in response units. At the end of each sample injection (120 sec), HBS-EP buffer was passed over the sensor surface to monitor the dissociation phase. The equilibrium dissociation constant (Kd) was derived by a steady state binding model.

### Immunostaining analysis

For immunocytochemistry, HUVECs were grown to confluence on glass coverslips coated with 2% gelatin and treated with or without VEGF-A (10 ng/ml) for 30 min, followed by incubation with FITC-RLYE (100 ng/ml). Cells were fixed in 3.7% formaldehyde for 30 min and permeabilized with 0.2% Triton X-100 in PBS, then incubated with blocking solution of PBS containing 3% normal goat serum and 0.05% Tween-20. Cells were labeled with antibodies against human VE-cadherin (Santa Cruz) and human CD31 (PECAM-1, Santa Cruz). Tumor sections (30 μm) were stained as previously described [45] by incubating with one of the following antibodies: rat anti-CD31, (Pharmingen), goat anti-VE-cadherin (Santa Cruze), and rabbit anti-NG2 (Millipore) for 2 h at room temperature, rinsed in PBS, and incubated with Alexa Fluor-, FITC- or TRITC-conjugated secondary antibody for 60 or 90 min at room temperature. Some tumor sections were also incubated with FITC-isolectin B4 (5 μg/ml; Vector Laboratories, Burlingame, CA) for 1 h. The sections were mounted with Permount solution after washing three times with PBS. Images were photographed and analyzed using a confocal fluorescence microscope. Nuclear staining was performed with 4’, 6-diamidino-2-phenylindole (DAPI, 1 ng/ml), and apoptotic cells were detected using a terminal deoxynucleotidyl transferase dUTP nick end labeling (TUNEL) kit (Roche, Korea).

### Docking simulations

Coordinates of RLYE peptide were generated by Chimera [47]. We performed blind docking of RLYE to VEGFR-2 (PDB ID 2×1W), which was extracted from the structure of the VEGF/VEGFR-2 complex [26]. Using Autodock 4.2 [48], a large grid box of 0.15 Å spacing was set up to encompass the whole VEGFR-2 molecule. Number of energy evaluations was increased to 50 million to account for the large number of rotatable bonds in the RLYE peptide. We performed 1000 blind docking simulations with a Lamarckian genetic algorithm. Chimera software was used for graphic presentation [47].

### Statistical analysis

Quantitative data are expressed as mean ± standard deviation (SD) of at least three separate experiments. Statistical significance was determined using the unpaired Student's *t* test or ANOVA, depending on the number of experimental groups analyzed. Significance was established at a *p* value <0.05.

## SUPPLEMENTARY MATERIALS FIGURES


