# Supplementary material for: Arg-Leu-Tyr-Glu tetrapeptide inhibits tumor progression by suppressing angiogenesis and vascular permeability via VEGF receptor-2 antagonism
Source: Oncotarget. 2016 Dec 28;8(7):11763–77. doi: 10.18632/oncotarget.14343 (PMC5355302; doi:10.18632/oncotarget.14343)
Supplement: Supplementary file 1 [file oncotarget-08-11763-s001.pdf]

Arg-Leu-Tyr-Glu tetrapeptide inhibits tumor progression by suppressing angiogenesis and vascular permeability via VEGF receptor-2 antagonism

SUPPLEMENTARY FIGURES

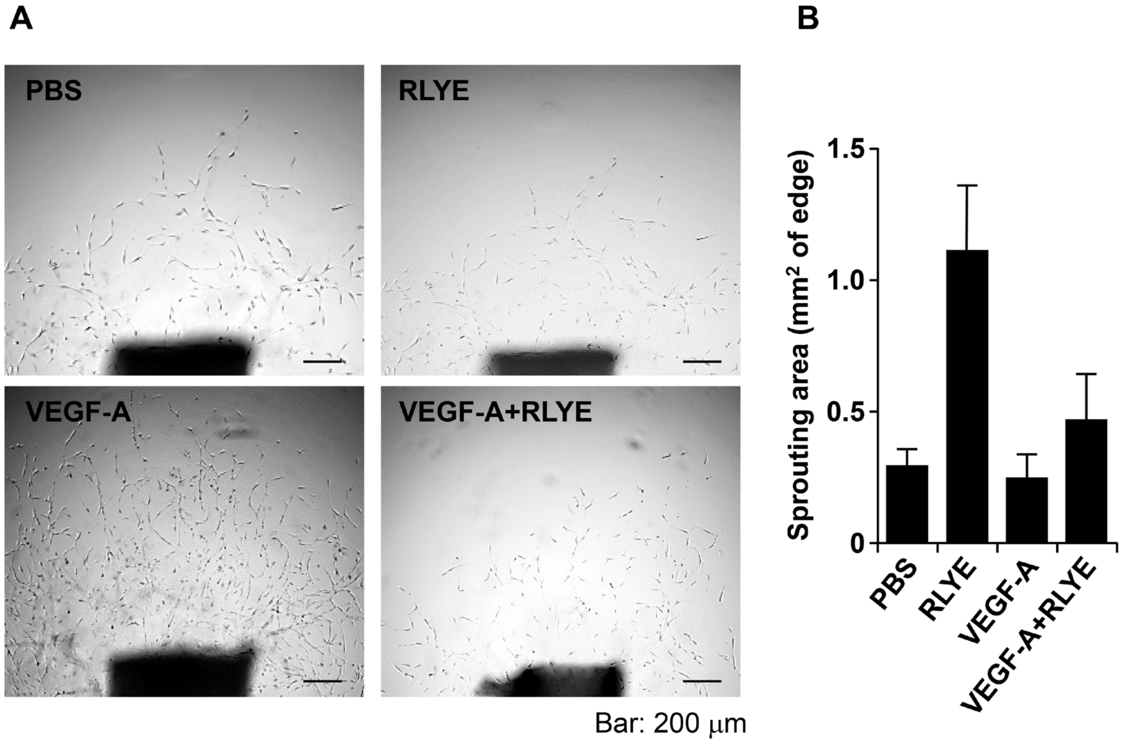

Supplementary Figure 1: RLYE inhibits VEGF-A-induced mouse aortic ring sprouting. Mouse aortic rings were incubated with VEGF-A, RLYE or combined together. **A.** On day 6, newly formed vessels were fixed and microvessel outgrowth was photographed under a phase contrast microscope. **B.** The area of sprouting per millimeter of tissue was quantified with Image J software (NIH; http://rsb.info.nih.gov/ij).

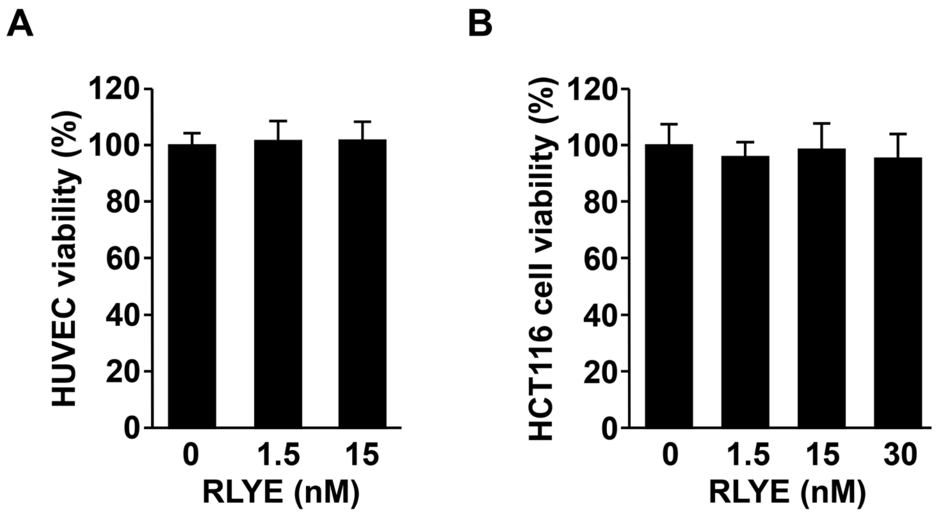

Supplementary Figure 2: RLYE does not exert any cytotoxicity on HUVECs and HCT116 cells. HUVECs **A.** and HCT116 **B.** cells were treated the indicated concentration of RLYE for 24 h. Cell viability was determined by MTT assay.

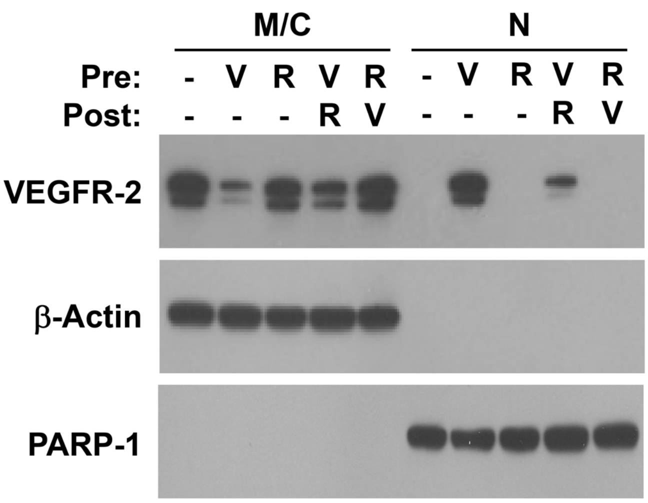

Supplementary Figure 3: RLYE inhibits VEGF-A-induced VEGFR-2 internalization. HUVECs were treated with RLYE alone, VEGF-A alone or both in combination for 30 min. Membrane/cytosolic (M/C) and nuclear (N) fractions were prepared, and Western blot was performed for determining VEGFR-2 levels in each fraction.
